# Ultrasound performed by emergency clinician improved the diagnostic efficacy in deep vein thrombosis

**DOI:** 10.1186/2036-7902-7-S1-A28

**Published:** 2015-03-09

**Authors:** Chien-Ming Chu, Kuo-Chih Chen, Tzong-Luen Wang

**Affiliations:** Emergency Department, Shin Kong Wu Ho-Su Memorial Hospital, Taipei, Taiwan

**Keywords:** Emergency Department, Pulmonary Embolism, Treatment Time, Deep Vein Thrombosis, Femoral Vein

## Background

Compression ultrasonography has proven to be a highly sensitive and specific modality for the recognition of lower extremity deep vein thrombosis (DVT). Traditionally, lower extremity studies are performed by an ultrasonography technologist. However, bedside ultrasound performed by well-trained emergency clinician may help to decrease the waiting time for diagnosis and treatment.

## Objective

To assess the efficacy of bedside ultrasound for the detection of deep vein thrombosis in the emergency department (ED).

## Patients and methods

From January 1^st^ 2006 to March 31th 2014, patients who were diagnosed as deep vein thrombosis, either by conventional duplex studies or bedside ultrasound, were included. Medical records were reviewed by a 6-year-trained emergency clinician. Age less than 18, patient who was referred from outpatient department or from other hospital, patient who had the diagnosis of deep vein thrombosis or pulmonary embolism before the visit, patient who use any anti-coagulant before the visit were excluded. Diagnosis of deep vein thrombosis was confirmed by any non-compressible venous vasculature of lower extremities, including common femoral vein, superficial and deep femoral vein, and popliteal vein, under ultrasound. Patient was divided into two group, one group was diagnosed by conventional duplex, and another group was diagnosed by bedside ultrasound. We compared the diagnostic time, treatment time, ED stay time between these two groups. Diagnostic time, treatment time, ED stay time were defined as time between the patient arrived ED and ultrasound-confirmed DVT, time between the patient arrived ED and patient first receive anti-coagulant, time between the patient arrived ED and patient admission or discharge, respectively.

## Results

Seventy-three patient were enrolled in this study, and 34 patients were male. The mean age was 60, ranging from 24 to 90. Sixteen patient had history of malignancy, 3 patient received major surgery within 3 months, and 2 patient had oral contraceptives. Bedside ultrasound significantly decreased the diagnostic time (2.24±0.43 hours and 17.28±4.77 hours, p< 0.001), treatment time (2.80±0.47 hours and 12.77±4.71 hours, P =0.001), ED stay time (13.49±2.80 hours and 31.74±6.89 hours, p =0.004).

## Conclusion

Bedside ultrasound decreased the diagnosis time, treatment time, and ED stay time, which may help to relief the ED crowding condition in a medical center.

